# Standardization of gesture apraxia assessment in the elderly population of Olinda city in Northeast Brazil

**DOI:** 10.1590/1980-5764-DN-2025-0336

**Published:** 2026-03-06

**Authors:** Paulo Roberto de Brito-Marques, Amanda Braga-Santos

**Affiliations:** 1 Universidade de Pernambuco, Faculdade de Ciências Médicas, Unidade de Neurologia Cognitiva e Comportamental, Recife PE, Brazil.; 2 Universidade de Pernambuco, Hospital Universitário Oswaldo Cruz, Recife PE, Brazil.; 3 Universidade de Pernambuco, Faculdade de Ciências Médicas, Pós-Graduação em Especialização em Neurologia Cognitiva e Comportamental, Recife PE, Brazil.

**Keywords:** Apraxia, Ideomotor, Neuropsychology, Cognition, Aging, Mental Status and Dementia Tests, Apraxia Ideomotora, Neuropsicologia, Cognição, Envelhecimento, Testes de Estado Mental e Demência

## Abstract

Apraxia is the inability to voluntarily perform learned gestures, even though sensory and motor functions are preserved.

**Objective:**

To standardize apraxia gestures and pantomimes in the elderly population.

**Methods:**

A brief clinical and family history, demographic data, the Edinburgh Handedness Inventory, the Mini-Mental State (MMS), the Modified Mini-Mental State (MMMS), and an evaluation of ideomotor apraxia and pantomimes based on Liepmann’s criteria were conducted.

**Results:**

There were significant associations between pantomime tasks and MMS, MMMS, and age. Additionally, significant correlations were observed between ideomotor apraxia and MMS, MMMS, age, and higher education levels.

**Conclusion:**

The gesture tests demonstrated neuropsychological potential applicability in elderly individuals aged 60 to 79 who have completed at least eight years of education.

## RESUMO.

Apraxia é a incapacidade de realizar gestos aprendidos voluntariamente com suas funções sensoriais e motoras preservadas.

**Objetivo:**

Padronizar gestos e pantomimas de apraxia na população idosa.

**Métodos:**

Foram realizados um breve histórico clínico e familiar, levantamento de dados demográficos, o Inventário de Dominância Lateral de Edimburgo, o Miniexame do Estado Mental (MEM), o Miniexame do Estado Mental Modificado (MEMM) e uma avaliação da apraxia ideomotora e das pantomimas com base nos critérios de Liepmann.

**Resultados:**

Encontraram-se associações significativas entre as tarefas de pantomima e o MEM, o MEMM e a idade. Além disso, foram observadas correlações significativas entre a apraxia ideomotora e o MEM, o MEMM, a idade e o nível de escolaridade superior.

**Conclusão:**

O teste de gestos práxicos apresenta potencial neuropsicológico para sua aplicação na população idosa de 60 a 79 anos com pelo menos oito anos de escolaridade.

## INTRODUCTION

 Daily routines consist of a series of movements, which can be classified into those that require tools and those that do not. Activities such as preparing meals and performing household chores belong to the first group, as they often involve the use of utensils and appliances. Actions such as getting dressed, cleaning oneself, and taking care of one’s appearance also require a sequence of steps and refined, coordinated movements of the upper limbs. It is undeniable that the ability to perform these activities daily is fundamental to an individual’s autonomy, regardless of their level of education^1^. 

 The term ‘praxis’ (from the Greek, meaning action) refers to the cognitive ability to perform an intentional, organized, and previously learned motor act with a specific purpose. Therefore, ‘apraxia’ is defined as an impairment in the correct execution of purposeful movements in the absence of weakness, involuntary movements, incoordination, or sensory alterations^2^. John Hughlings Jackson first clearly described apraxia in 1861, although he did not provide the specific term^3^. Apraxias are subtle motor disorders characterized by a disruption in the organization of movement, typically located in the left hemisphere. It was not until 1871 that Heymann Steinthal, a German linguist, introduced the term apraxia to describe the incorrect use of objects in everyday life^4^. 

 However, Hugo Karl Liepmann was credited with its elucidation^5^. He proposed that, for right-handed individuals, the central role of the parietal lobe in apraxia, and Kenneth Heilman emphasized the left inferior parietal lobe as the center of *praxicons*. On the other hand, Liepmann suggested that the left cerebral hemisphere (LCH) contained motor representations, or movement formulas that specified the spatial and temporal characteristics of skilled and intentional movements^6^. 

 Liepmann described three forms of limb apraxia: limb-kinetic, ideomotor, and ideational. Since his time, advances in behavioral neurology, neuropsychology, and neuroimaging have enhanced our understanding of the nature and manifestations of limb apraxia. However, the original terminology and much of his theory regarding the mechanisms of apraxia remain relevant today^7-11^. Ideomotor apraxia (IMA) is a disorder in which manual gestures (ideomotor apraxia for intransitive gestures) or the use of a tool (ideomotor apraxia for transitive gestures) cannot be performed upon verbal command, despite intact task knowledge^12-14^. Ideational apraxia occurs due to the inability to conceptualize a task, leading to errors in the sequence of multistep actions rather than in a single movement, despite intact tool identification^15-17^. Kinetic apraxia of the limbs, or melokinetics, is characterized by the loss of the ability to make precise, independent, and coordinated movements of the fingers and hands, resulting in imprecise or clumsy movements^18^. 

 The definitions of apraxia were later expanded and clarified by Norman Geschwind’s concept of disconnection syndromes, described in the 1960s^19^. According to Geschwind, when a patient receives a command to perform a gesture, the verbal message is decoded in Wernicke’s area. This message must then be transmitted to the left premotor convexity cortex, so that the left primary motor cortex can execute the command. The premotor cortex of the left cerebral hemisphere connects asymmetrically with the primary motor cortex. Therefore, when attempting to perform verbal commands, a disconnection between the premotor cortex and Wernicke’s area would prevent the premotor cortex in both hemispheres from receiving instructions about the requested movement^20^. Gestures are acquired and learned throughout life, influenced by variations in environments and cultures. From a neuropsychological perspective, IMA and conceptual apraxia models can be used in the elderly population to detect signs consistent with early-stage Alzheimer’s disease^21-23^. Our objective was to standardize gestures in the elderly population in Northeastern Brazil and compare them to already established models. 

## METHODS

### Design

 The study was a cross-sectional, randomized sample of 276 participants living in Olinda city, state of Pernambuco, Brazil. 

 The sample consisted of 276 individuals, 73% of whom were female, and was based on the following data:

1. The neighborhood’s streets were listed and drawn using an updated map.
2. On each street, all participants aged between 60 and 92 were tested, complying with the research inclusion criteria.

The sample size was calculated based on a pilot observation, a population standard deviation (SD) of 3.8, a confidence interval of 95%, and a precision of 0.5. Besides a margin of 3%, they were preventing a possible drop-out. The population consisted of 276 elderly people aged between 60 and 89 years. The average age was 69.4 (± 6.8 years SD); 73.9% were females. According to Oldfield^24^, of the total sample, 98.5% were right-handed. Participants were stratified by age into six subgroups: 60–64, 65–69, 70–74, 75–79, 80–84, and 85–89. The average level of schooling was four years. Schooling was categorized into four subgroups: illiterate or zero, between one and four years, between five and eight years, and more than eight years. Cardiovascular risk factors were found in the personal history, in addition to the history of depression. All subjects underwent an assessment of age, schooling, the Mini-Mental State (MMS)^25^, and the Modified Mini-Mental State (MMMS)^26,27^ tests. The MMMS is a test conducted in Northeast Brazil to assess the cognitive function of the elderly, taking their level of schooling into account. The average level of MMS was 23.8, and the average level of MMMS was 25.5, respectively. The local ethics committee approved the research, under Certificate of Presentation for Ethical Appreciation (CAAE): 39103420.1.0000.5192. The confidentiality of the study was guaranteed by those responsible for the participants’ records. Exclusion criteria were uncorrected visual and auditory deficits, neurological and psychiatric disorders, or joint disease that hindered motricity, and participants with intellectual disabilities were differentiated from illiterates by not recognizing colors, use of money, and how to use a can opener. Individuals with dementia were not included in the study. 

### Procedure

 According to Signoret^28^, in the test to assess pantomimes, the examiner asks the individual to obey their verbal command. The individual must make gestures per the orders only with their dominant hand (block A) and simultaneously in the second moment with both hands (block B). The individual performs the following monomanual gestures: how you drink a glass of water, how you brush your teeth, how you use a comb, dial a telephone, and how you use a key. Then, the individual performs the following bimanual gestures: how you iron clothes, how you hit a nail with a hammer, how you saw a piece of wood, how you look with binoculars, and how you fasten the necklace around your neck. 

 To assess ideomotor praxis, the examiner asks the individual to pay attention because they are in opposite positions^29^. The examiner sitting in front of the individual asks him to imitate him, only using his dominant hand (block A). The gestures performed by the examiner are: the back of the right hand over the left ear, Luria’s motor triad^30^, the palm of the right hand over the right ear, fist under the chin, right palm on the left shoulder blade and left palm on the head. Then the individual performs the following bimanual gestures: reciprocal coordination, back of the left hand and palm of the right hand outwards, left hand vertical and right hand horizontal, left hand over the right hand, touching the scapulas with the arms crossed^29^. 

 There is no set time for carrying out both praxis tests; however, if the patient takes longer than immediately, it should not exceed 30 seconds per gesture. 

### Scores

 Pantomime performance was scored on a scale from 0 to 10 points, with 5 points allocated to monomanual gestures and 5 to bimanual gestures. Errors were categorized as parapraxis, mirroring, or omission. To score the assessment of ideomotor praxis, the individual can score between 0 and 10 points, with 5 being monomanual and 5 being bimanual; errors can be parapraxis, mirroring, or unrealized^29^. One point was assigned for each correct item in the pantomime and ideomotor apraxia tasks, zero points for incorrect ones (Optimal Neuropsychological Evaluation of Dementias of Montreal, 1993). 

### Statistical analysis

 Descriptive statistics were initially calculated for all variables. In addition to Pearson’s correlation, multiple linear regression models were conducted to better control for potential confounding factors. The dependent variables were the total scores obtained in the pantomime and ideomotor praxis tests. Independent variables included age (continuous), while sex, education (categorized into four levels: illiterate, 1–4 years, 5–8 years, and >8 years), and cognitive performance (MMS, and MMMS) were entered as covariates. This approach allowed the estimation of the association between age and praxis performance while adjusting for sex, education, and cognitive status. Regression coefficients (β), confidence intervals of 95%, and p-values < 0.05 were considered statistically significant. All analyses were performed using The Statistical Package for the Social Sciences (SPSS) version 20.0. 

## RESULTS

 A predominance of females (73%) was observed, which may introduce bias into the results due to the gender imbalance. Results for pantomimes are presented. No significant result was found regarding education. However, a correlation was observed between the monomanual and bimanual pantomime results, age, and the MMS and MMMS tests. The correlation was negative and had a lower coefficient. The p-value was low, indicating statistical significance. The chart shows that, as MMS and MMMS scores increase, age decreases (Tables 1 and 2). 

**Table 1:** Shows the results of blocks A and B pantomime compared to the age and the Mini-Mental State test.

	R	p	Regression equation
Errors A	-0.17187	<0.001	Y=0.87645–0.02673*X
Errors B	-0.23279	<0.001	Y=1.86171–0.05620*X

Note: Pearson correlation.

**Table 2:** Shows the results of blocks A and B pantomime compared the age and the Modified Mini-Mental State test.

	R	p	Regression equation
Errors A	-0.2134	<0.001	Y=1.07226–0.03211*X
Errors B	-0.25952	<0.001	Y=2.102103–0.06062*X

Note: Pearson correlation.

 There was no overall correlation between the results of monomanual and bimanual ideomotor apraxia and either the level of education or MMS test scores. However, after stratifying for education and comparing the results from Blocks A and B with MMS scores, participants with more than eight years of schooling demonstrated a significant reduction in errors, with statistically significant findings in both blocks. The correlation was negative, with a low coefficient, and the p-value was also low, confirming statistical significance. The chart demonstrates a decrease in errors with increasing MMS scores, especially in Block A (Table 3). 

**Table 3:** Shows the results of ideomotor apraxia, education level, and the Modified Mini-Mental State test.

	R	p	Regression equation
Errors A	-0.14534	0.01549	Y=1.66212–0.05947*X
Errors B	-0.10959	0.06857	Y=1.9327–0.05182*X

Note: Pearson correlation.

 A correlation was observed between blocks A and B of ideomotor apraxia and age. A positive but low correlation coefficient was found, with a low p-value, indicating statistical significance. The chart shows evidence that, as age increases, the number of errors also increases (Table 4). 

**Table 4:** Shows the results of ideomotor apraxia compared to age.

	R	p	Regression equation
Errors A	0.13884	0.0208	Y=-0.4107+0.0262*X
Errors B	0.10478	0.08172	Y=0.12509+0.02285*X

Note: Pearson correlation.

 A correlation was performed between blocks A and B of ideomotor apraxia and the MMS test. A negative moderate correlation coefficient was found, with a low p-value, indicating statistical significance. The chart shows evidence that, as the MMS score increases, the number of errors decreases (Table 5). 

**Table 5:** Shows the results of ideomotor apraxia compared to the Mini-Mental State test.

	R	p	Regression equation
Errors A	-0.37094	<0.001	Y=4.02489–0.11617*X
Errors B	-0.25087	<0.001	Y=3.75654–0.09079*X

Note: Pearson correlation.

 A correlation was performed between blocks A and B of ideomotor apraxia and the MMMS test. Chart evidence shows that that, if the MMMS increases, the errors decrease (Table 6). 

**Table 6:** Shows the results of ideomotor apraxia compared to the Modified Mini-Mental State test.

	R	p	Regression equation
Errors A	-0.38091924	<0.001	Y=4.27614–0.11543*X
Errors B	-0.24005369	<0.001	Y=3.80009–0.08407*X

Note: Pearson correlation.

 A correlation analysis was performed between ideational apraxia according to age group (Table 7). 

**Table 7:** Shows results between ideational apraxia according to age group.

	r	p	Regression equation
60–64	0.01376	0.90669	Y=0.6355+0.01122*X
65–69	0.10423	0.33104	Y=-4.78674+0.08818*X
70–74	-0.11917	0.39074	Y=10.72911–0.12506*X
75–79	0.19250	0.29117	Y=-12.85252+0.18784*X
80–84	0.23158	0.38813	Y=-18.89873+0.24684*X
more than 84	0.02067	0.95190	Y=0.97021+0.01489*X

Note: Pearson correlation.

## DISCUSSION

 Elderly individuals from a community were studied to observe the performance of voluntary gestures corresponding to pantomimes and ideomotor praxis, using the right hand, and both hands. These were compared with age, education, and the MMS and MMMS tests. It was observed that the elderly group did not show significant results when comparing age and education with pantomimes. The relationship between the sexes was not studied. 

 Pantomime is used in clinical neuropsychology as a cognitive process based on the gesture-engram hypothesis, and tool use is considered a communicative gesture rather than a replication of the motor action’s real use^31-35^. It’s possible that age and education did not interfere with the performance of pantomimes in our elderly individuals due to the strong cultural influence in Northeast Brazil^29,36^. However, some symbolic gestures may facilitate the pantomime performance^37-40^. 

 The increase in education is proportional to the results of MMMS^26^. Limb ideomotor apraxia is a disorder affecting learned skilled movements and the ability to imitate gestures while performing practical tasks throughout life, which depends on good perception and motor skills, requiring a higher level of education^14^. Elderly individuals with little or no education may struggle to perform practical tasks with dexterity, making errors such as parapraxia, mirror movements, or failing to complete the tasks altogether. 

 IMA is generally less severe with intransitive than transitive gestures and improves with imitation and the use of actual tools or objects^2,6^. There is a tendency for increased difficulty in performing IMA tasks in daily life activities as part of the aging process^14,31,32^. On the other hand, in this study, ideational apraxia showed similar results across all age groups (Table 7). However, other factors, such as culture, education level, and asymptomatic neurodegenerative diseases, may also potentially affect praxis performance^38,39^. Gestural performance is strongly influenced by the left hemisphere of the brain, with language playing a central role^8^. Public basic education in Northeast Brazil requires improvement in analytical knowledge, as individuals may experience further decline as they age^26,27^. 

 The MMS is the most widely used clinical test for screening mild cognitive impairment and dementia^26-28,31^. However, it is also commonly used in comparison with neuropsychological tests^41-45^. We observed a correlation between the MMS and MMMS compared to apraxia. The results showed a moderate negative correlation coefficient, with a low p-value indicating statistical significance. The chart also demonstrated that, as scores on both tests increased, the number of apraxia errors decreased. 

 In conclusion, we present a population-based study on gesture apraxia, conducted in the city of Olinda, Brazil. The study included older adults aged 60 to 90, stratified by education level. Gesture performance was analyzed according to age, education, and scores on the MMS and MMMS. The findings revealed that the oldest participants, those over 80 years, had greater difficulty performing gestures, particularly individuals with less than eight years of education. We suggest that many elderly individuals may experience impaired sensory perception during gesture-related tasks in the context of apraxia. Pantomime performance may also have been affected by disruptions in manipulation knowledge and by low levels of education^45^. Additionally, cultural factors may have contributed to the outcomes observed. 

 The main limitation of this study was the lack of more detailed control over the influence of different cultural backgrounds. 

## Data Availability

The datasets generated and/or analyzed during the current study are not publicly available due to [ethical/legal/privacy] restrictions but are available from the corresponding author upon reasonable request.
